# Blood urea nitrogen to serum albumin ratio as a new prognostic indicator in type 2 diabetes mellitus patients with chronic kidney disease

**DOI:** 10.1038/s41598-024-58678-4

**Published:** 2024-04-05

**Authors:** Shizhen Liu, Chuangye Qiu, Wenxia Li, Xingai Li, Fanna Liu, Guoqiang Hu

**Affiliations:** 1 Department of Nephrology, Jiangmen Central Hospital, Jiangmen, Guangdong China; 2 Department of Endocrinology, Jiangmen Central Hospital, Affiliated Jiangmen Hospital of Sun Yat-Sen University, Jiangmen, Guangdong China; 3 Department of Nephrology, The First Affiliated Hospital of Jinan University, Guangzhou, 510630 Guangdong China

**Keywords:** Type 2 diabetes mellitus, Chronic kidney disease, Intensive care unit, Mortality, Blood urea nitrogen to serum albumin ratio, Biomarkers, Diseases, Endocrinology, Health care, Nephrology, Risk factors

## Abstract

Chronic kidney disease (CKD) is often a common comorbidity in critically ill patients with type 2 diabetes mellitus (T2DM). This study explored the relationship between blood urea nitrogen to serum albumin ratio (BAR) and mortality in T2DM patients with CKD in intensive care unit (ICU). Patients were recruited from the Medical Information Mart database, retrospectively. The primary and secondary outcomes were 90-day mortality, the length of ICU stay, hospital mortality and 30-day mortality, respectively. Cox regression model and Kaplan–Meier survival curve were performed to explore the association between BAR and 90-day mortality. Subgroup analyses were performed to determine the consistency of this association. A total of 1920 patients were enrolled and divided into the three groups (BAR < 9.2, 9.2 ≤ BAR ≤ 21.3 and BAR > 21.3). The length of ICU stay, 30-day mortality, and 90-day mortality in the BAR > 21.3 group were significantly higher than other groups. In Cox regression analysis showed that high BAR level was significantly associated with increased greater risk of 90-day mortality. The adjusted HR (95%CIs) for the model 1, model 2, and model 3 were 1.768 (1.409–2.218), 1.934, (1.489–2.511), and 1.864, (1.399–2.487), respectively. Subgroup analysis also showed the consistency of results. The Kaplan–Meier survival curve analysis revealed similar results as well that BAR > 21.3 had lower 90-day survival rate. High BAR was significantly associated with increased risk of 90-day mortality. BAR could be a simple and useful prognostic tool in T2DM patients with CKD in ICU.

## Introduction

The international diabetes federation predicted that the global population of diabetes will reach to 454 million in 2030; furthermore, the number will increase to 783 million in 2045^1^. Type 2 diabetes mellitus (T2DM) and chronic kidney disease (CKD) are both chronic diseases. A study showed that the prevalence of T2DM patients complicated with CKD reached to 64–81.6/10,000, and it will be further increased^2^. CKD is one of the most debilitating and expensive complication of T2DM^3^. T2DM complicated with CKD will reduce life expectancy by 16 years than without CKD, which will increase higher medical cost burden and mortality^4,5^. T2DM and CKD patients often need to restrict their diet during their daily treatment, and thus are prone to disease-related malnutrition, which further leads to poor prognosis before admission to ICU^6,7^.

T2DM patients with CKD are accompanied by protein-energy malnutrition and inflammation before admission to ICU. Blood urea nitrogen (BUN) not only reflects the status of kidney function, but also as an important indicator of protein nutrient metabolism level and inflammation^8^. A study found that high BUN levels were positively associated with increased adverse renal outcomes^9^. Albumin is the main component of plasma proteins, and as a commonly used biomarker in clinical practice, its slight changes can affect the prognosis of patients in various diseases. A study showed that low serum albumin levels accelerated the decline of kidney function^10^. Studies have reported that BAR can be used as a useful biomarker for poor prognosis of many diseases, such as gastrointestinal bleeding, sepsis, COVID-19, and acute kidney injury^11–13^.

However, since the impact of BAR among T2DM patients with CKD has not yet been investigated, in the study, we evaluated the relationship between BAR and the prognosis of T2DM patients with CKD in ICU.

## Materials and methods

### Data source

This was a retrospective cohort study that involved Medical Information Mart for Intensive Care III (MIMIC-III) (https://physionet.org/content/mimiciii/1.4/) database. MIMIC-III database contains clinical information on over 50,000 ICU patients at the Beth Israel Deaconess Medical Center from 2001 to 2012^14^. After completing the online training course of the National Institutes of Health, the author (S.L.) obtained access to this database (certification number: 42883491). Accessed to database was approved by the Institutional Review Boards of Beth Israel Deaconess Medical Center (Boston, MA) and the Massachusetts Institute of Technology (Cambridge, MA). We extracted patient demographics, laboratory findings, length of hospital stay, 30- and 90-day mortality, and other clinical variables by PostgreSQL V.10.0. Our study was performed in accordance with the Declaration of Helsinki (as revised in 2013).

### Inclusion and exclusion criteria

**Figure 1:**
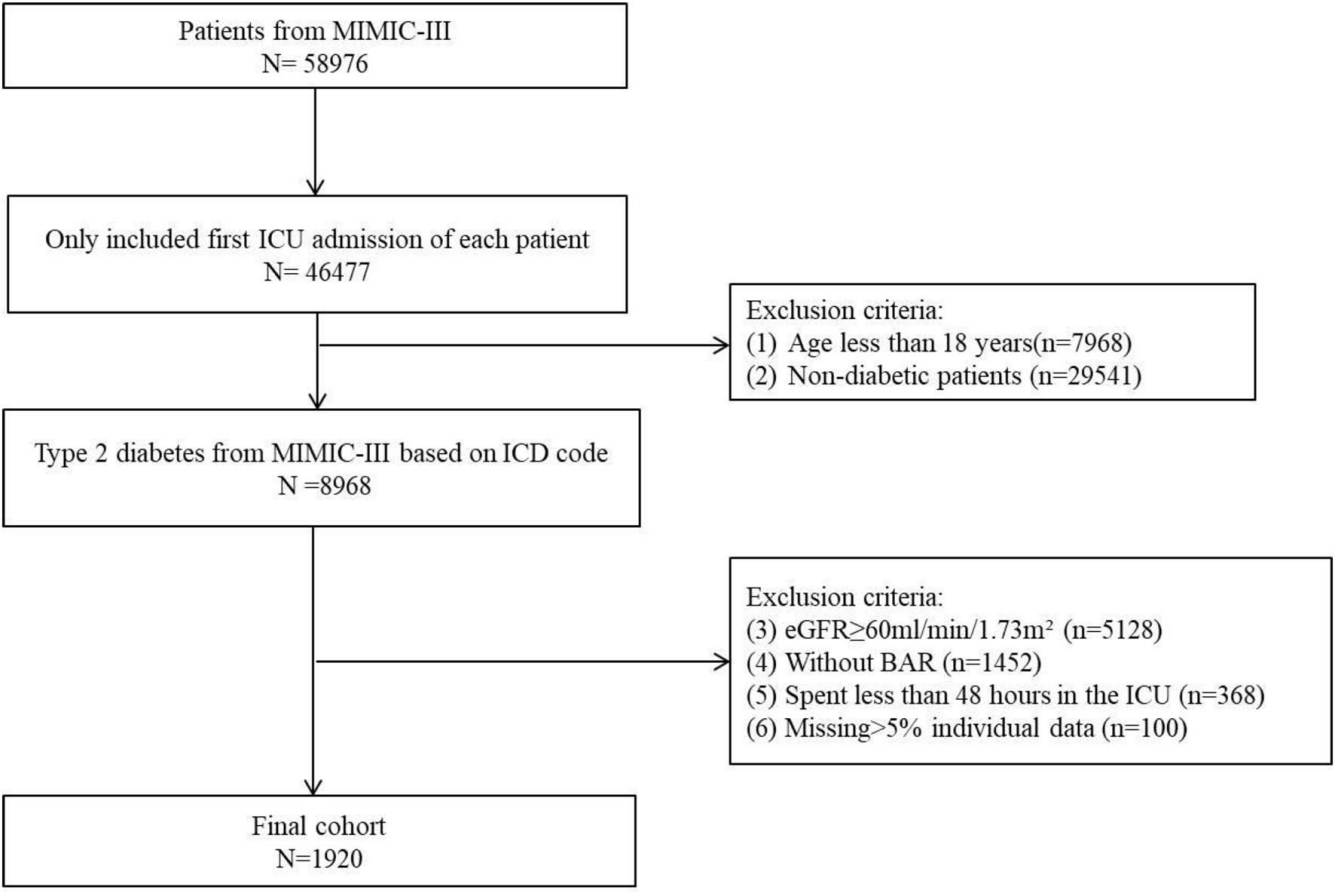
Flow diagram of the study.

Patients admitted to the ICU for the first time were included in this study. Patients were excluded according to: (1) Less than 18 years old; (2) ICD code is not T2DM. (3) Without CKD. According to the KDIGO clinical practice guidelines, CKD was diagnosed that glomerular filtration rate (GFR) below 60 mL/min/1.73 m^2^ for 3 months or more^15^. (4) Missing BUN or serum albumin values; (5) Less than 48 h in ICU; (6) Missing data for more than 5% of patients. Finally, a total of 1920 patients were included in this study (Fig. 1). We had listed the top five diagnosed diseases and top five diseases that were first diagnosed in this population at admission to ICU (Supplementary Table 1, 2).

### Data extraction

We extracted the variables as follows: age, gender, weight, systolic blood pressure (SBP), diastolic blood pressure (DBP), coronary artery disease (CAD), chronic heart failure (CHF), hypertension, CKD stage, sequential organ failure assessment (SOFA), hemoglobin (HGB), white blood cell (WBC) count , platelet count, potassium, sodium, phosphate, creatinine, blood urea nitrogen (BUN), albumin (ALB), alkaline phosphatase (ALP), alanine transaminase (ALT), aspartate transaminase (AST), glucose, lactate level, arterial oxygen partial pressure (PaO_2_), partial thromboplastin time (PTT), prognostic nutritional index (PNI) and estimate-GFR (eGFR) by structured query language in PostgreSQL. We had listed some diseases that might influence the prognosis and clinical data itself (Supplementary Table 3). BAR was calculated by dividing the BUN by the albumin.

### Statistical analysis

Patients were divided into three groups: BAR < 9.2, 9.2 ≤ BAR ≤ 21.3 and BAR > 21.3, according to the interquartile ranges (IQRs) of BAR value. Continuous variables were presented as mean ± standard deviations or IQRs, and classification variables were presented as totals and percentage (%). The chi-square test was used for classified variables between groups. The Wilcoxon rank-sum test was used for non-normally distributed continuous variables, and student t-test was used for normally distributed continuous variables. Univariate and multivariate cox regression were used to assess the independent factors associated with 90-days mortality, which presented as the hazard ratio (HR) and 95% confidence interval (CI). In order to reduce the impact of confounding factors, we constructed three Cox regression models to identify the potential clinical usefulness of BAR by including covariates with *p* values < 0.05 in the univariate Cox analyses or for importance of clinical concern. Kaplan–Meier survival analysis was used to determine the difference in 90-day mortality between the three groups. Stratification analyses was used to assess the association of BAR with 90-day mortality. *P* < 0.05 was considered statistically significant. The statistical analyses were performed using the Stata software version 16.0 (Stata Corp. LLC, TX, US).

### Ethical approval and consent to participate

The informed consent was waived by the Institutional Review Boards of Beth Israel Deaconess Medical Center (Boston, MA) and the Massachusetts Institute of Technology (Cambridge, MA). The patient's information has been standardized and the project did not affect clinical care, so requirement for individual patient consent was waived.

## Results

### Baseline characteristics

A total of 1920 patients were enrolled and were divided into the three groups: low-BAR group (BAR < 9.2, n = 486), mid-BAR group (9.2 ≤ BAR ≤ 21.3, n = 954), and high-BAR group (BAR > 21.3, n = 480). The number of man was significantly higher in the high-BAR group with lower proportion of hypertension. A higher proportion of CHF, CKD 4 stage and CKD 5 stage, along with higher levels of SOFA score, WBC, potassium, phosphate, creatinine, ALP, lactate and PTT in the high-BAR group; Patients with higher BAR had lower age, SBP, DBP, HGB, sodium, PaO_2,_ PNI, and eGFR (all *p* < 0.05) (Table 1).

**Table 1:** Comparisons of demographics within three BAR levels.

Variable	BAR < 9.2 (n = 486)	BAR 9.2–21.3 (n = 954)	BAR > 21.3 (n = 480)	*p* value
Age (years)	79 (70, 85)	75 (66, 82)	72 (63, 80)	< 0.001
Male, n(%)	197 (40.5)	508 (53.2)	284 (59.2)	< 0.001
Weight (kg)	70.0 (60.0, 80.6)	78.3 (67.0, 90.4)	85.0 (72.0, 97.4)	< 0.001
SBP(mmHg)	133 (111, 151)	124 (106, 145)	120 (103, 137)	< 0.001
DBP(mmHg)	62 (51, 75)	59 (50, 70)	56 (47, 68)	< 0.001
Comorbidities, n (%)				
CAD	166 (34.2)	327 (34.3)	142 (29.6)	0.172
CHF	178 (36.6)	450 (47.2)	247 (51.5)	< 0.001
Hypertension	264 (54.3)	330 (34.6)	115 (24.0)	< 0.001
GI bleeding	36	58	42	0.443
CKD stage, n(%)				< 0.001
3	406 (83.5)	591 (61.9)	176 (36.7)	
4	58 (11.9)	257 (26.9)	205 (42.7)	
5	22 (4.6)	106 (11.2)	99 (20.6)	
SOFA score	4.0 (2.0,6.0)	6.0 (4.0,8.0)	6.0 (4.0,9.0)	< 0.001
Laboratory tests				
HGB (× g/L)	10.8 (9.6, 12.1)	10.2 (9.1, 11.4)	9.6 (8.7, 10.9)	< 0.001
Platelet (× 10^9^/L)	209.0 (153.0, 270.0)	200.0 (144.0, 272.0)	203.5 (139.5, 271.0)	0.441
WBC (× 10^9^/L)	10.8 (7.9, 14.3)	11.2 (7.9, 16.0)	11.9 (8.2, 16.7)	0.008
Potassium (mmol/L)	4.0 (3.7, 4.5)	4.4 (3.8, 4.9)	4.5 (4.0, 5.2)	< 0.001
Sodium (mmol/L)	139.0 (136.0, 141.0)	138.0 (135.0, 141.0)	138.0 (134.0, 141.0)	0.002
Phosphate (mg/dL)	3.4 (2.8, 4.1)	4.0 (3.2, 5.0)	4.9 (3.8, 6.4)	< 0.001
Creatinine (mg/dL)	1.3 (1.0, 1.5)	2.0 (1.5, 2.9)	3.1 (2.1, 4.8)	< 0.001
BUN (mg/dL)	22.0 (18.0, 27.0)	41.0 (34.0, 51.0)	77.0 (63.0, 96.0)	< 0.001
ALB (g/dl)	3.4 (2.9, 3.7)	2.9 (2.6, 3.4)	2.7 (2.3, 3.1)	< 0.001
ALP (U/L)	95.5 (68.0, 118.0)	104.0 (68.0, 120.0)	107.0 (73.0, 130.0)	0.007
ALT (IU/L)	33.0 (17.0, 77.0)	32.5 (17.0, 77.0)	32.0 (17.0, 77.0)	0.624
AST (IU/L)	45.0 (25.0, 136.0)	51.0 (25.0, 136.0)	47.0 (23.0, 136.0)	0.379
Glucose(mg/dL)	153.0 (120.0, 201.0)	156.5 (119.0, 216.0)	162.0 (113.0, 221.0)	0.779
Lactate (mmol/L)	2.4 (1.6, 2.4)	2.4 (1.5, 2.4)	2.3 (1.3, 2.4)	0.023
PaO_2_ (mmHg)	155.0 (99.0, 176.0)	155.0 (89.0, 155.0)	130.0 (78.0, 155.0)	< 0.001
PTT (s)	30.6 (26.3, 38.7)	33.0 (28.2, 40.8)	33.9 (28.5, 43.8)	< 0.001
PNI	34.0 (29.0, 37.0)	29.0 (26.0, 34.0)	27.0 (23.0, 31.0)	< 0.001
eGFR (ml/min/1.73m^2^ )	45.0 (36.5, 52.9)	34.2 (23.6, 44.3)	24.2 (16.4, 35.1)	< 0.001

BAR, blood urea nitrogen to albumin ratio; SBP, systolic blood pressure; DBP, diastolic blood pressure; CAD, coronary artery disease; CHF, chronic heart failure; GI, gastrointestinal; CKD, chronic kidney disease; SOFA, sequential organ failure assessment; HGB, hemoglobin; BUN, blood urea nitrogen; ALB, albumin; ALP, alkaline phosphatase; ALT, alanine transaminase; AST, aspartate transaminase; PTT, partial thromboplastin time; PNI, prognostic nutritional index; eGFR, estimated GFR.

### BAR levels and outcome

Compared with low-BAR group (BAR < 9.2), patients with higher BAR had significantly higher the length of stay, in-hospital mortality, 30-day mortality and 90-day mortality (*p* < 0.05) (Table 2).

**Table 2:** BAR level and clinical outcome.

Clinical outcomes	BAR			
	< 9.2(n = 486)	9.2–21.3 (n = 954)	> 21.3 (n = 480)	*P* value
Length of stay (days)	2.8 (1.5, 4.9)	3.2 (1.9, 6.6)	4.0 (2.0, 8.5)	< 0.001
Hospital mortality (n, %)	60 (12.3)	141 (14.8)	105 (21.9)	< 0.001
30-day mortality (n, %)	93 (18.5)	227 (23.8)	149 (31.0)	< 0.001
90-day mortality (n, %)	122 (25.1)	292 (30.6)	193 (40.2)	< 0.001

### Association between the BAR and 90-day mortality

Univariate and multivariate Cox regression analyses were utilized to evaluate the significance of BAR in predicting 90-day mortality. Univariate regression analysis showed that age, weight, SBP, DBP, CAD, CKD stage, SOFA score, WBC, phosphate, lactate, PTT, eGFR, BAR, mid-BAR and high-BAR were significantly associated with 90-day mortality (*p* < 0.05) (Table 3). Unadjusted in model 1, high BAR level was significantly associated with higher risk of 90-day mortality (BAR as continuous variable, HR 1.018, 95% CI 1.011–1.027; mid-BAR, HR 1.261, 95% CI 1.021–1.557; high-BAR, HR 1.768, 95% CI 1.409–2.218). Adjust for age, gender, weight, SBP, DBP, CAD, CHF, hypertension, CKD stage and SOFA score in model 2, high BAR level was also significantly associated with higher risk of 90-day mortality (BAR as continuous variable, HR 1.021, 95% CI 1.012–1.030; mid-BAR, HR 1.304, 95% CI 1.043–1.631; high-BAR, HR 1.934, 95% CI 1.489–2.511). Furthermore, Adjust for model 2 plus HGB, WBC, platelet, potassium, sodium, phosphate, ALP, ALT, AST, glucose, PaO_2_, PTT and eGFR in model 3, high BAR level remained a greater risk of 90-day mortality (BAR as continuous variable, HR 1.018, 95% CI 1.009–1.028; mid-BAR, HR 1.277, 95% CI 1.011–1.618; high-BAR, HR 1.864, 95% CI 1. 399–2.487) (all *p* < 0.05) (Table 4).

**Table 3:** Univariate Cox regression analyses to assess risk factors associated with 90-day mortality in T2DM patients with CKD.

	HR(95%CI)	*P* value
Age	1.026 (1.018–1.034)	< 0.001
Gender	0.871 (0.742–1.021)	0.089
Weight	0.994 (0.990–0.998)	0.005
SBP	0.995 (0.993–0.998)	0.002
DBP	0.995 (0.990–0.999)	0.040
CAD	0.792 (0.665–0.943)	0.009
CHF	1.125 (0.960–1.319)	0.147
Hypertension	0.955 (0.809–1.128)	0.588
GI bleeding	1.116 (0.970–1.232)	0.347
CKD stage	1.121 (1.006–1.250)	0.039
SOFA score	1.099 (1.074–1.124)	< 0.001
HGB	0.979 (0.937–1.024)	0.353
WBC	1.028 (1.016–1.040)	< 0.001
Platelet	1.000 (0.999–1.001)	0.118
Potassium	0.964 (0.879–1.058)	0.439
Sodium	1.002 (0.988–1.016)	0.801
Phosphate	1.049 (1.001–1.099)	0.045
ALP	1.002 (1.001–1.003)	< 0.001
ALT	1.000 (0.999–1.001)	0.353
AST	1.000 (0.999–1.009)	0.120
Glucose	0.999 (0.998–1.000)	0.168
Lactate	1.089 (1.045–1.135)	< 0.001
PaO_2_	0.999 (0.998–1.000)	0.081
PTT	1.003 (1.000–1.006)	0.017
eGFR	0.990 (0.984–0.995)	< 0.001
BAR	1.018 (1.011–1.027)	< 0.001
BAR < 9.2	Ref	–
9.2 ≤ BAR ≤ 21.3	1.261 (1.021–1.557)	0.032
BAR > 21.3	1.768 (1.409–2.218)	< 0.001

**Table 4:** Association between BAR and 90-day mortality.

Variables	Model 1		Model 2		Model 3	
	HR (95% CI)	*P* value	HR (95% CI)	*P* value	HR (95% CI)	*P* value
BAR (continuous variable)	1.018(1.011–1.027)	< 0.001	1.021(1.012–1.030)	< 0.001	1.018(1.009–1.028)	< 0.001
BAR < 9.2	Ref	–	Ref	–	Ref	–
9.2 ≤ BAR ≤ 21.3	1.261 (1.021–1.557)	0.032	1.304 (1.043–1.631)	0.020	1.277 (1.011–1.618)	0.040
BAR > 21.3	1.768 (1.409–2.218)	< 0.001	1.934 (1.489–2.511)	< 0.001	1.864 (1.399–2.487)	< 0.001

BAR, blood urea nitrogen to albumin ratio; Ref, reference; HR, hazard ratio; CI, confidence interval. Model 1: unadjusted. Model 2: adjust for: age; gender; weight; SBP; DBP; CAD; CHF; hypertension; GI bleeding; CKD stage and SOFA score. Model 3; adjust for: Model 2 plus HGB; WBC; platelet; potassium; sodium; phosphate; ALP; ALT; AST; glucose; lactate; PaO_2_; PTT and eGFR.

### Prediction of 90-day mortality

**Figure 2:**
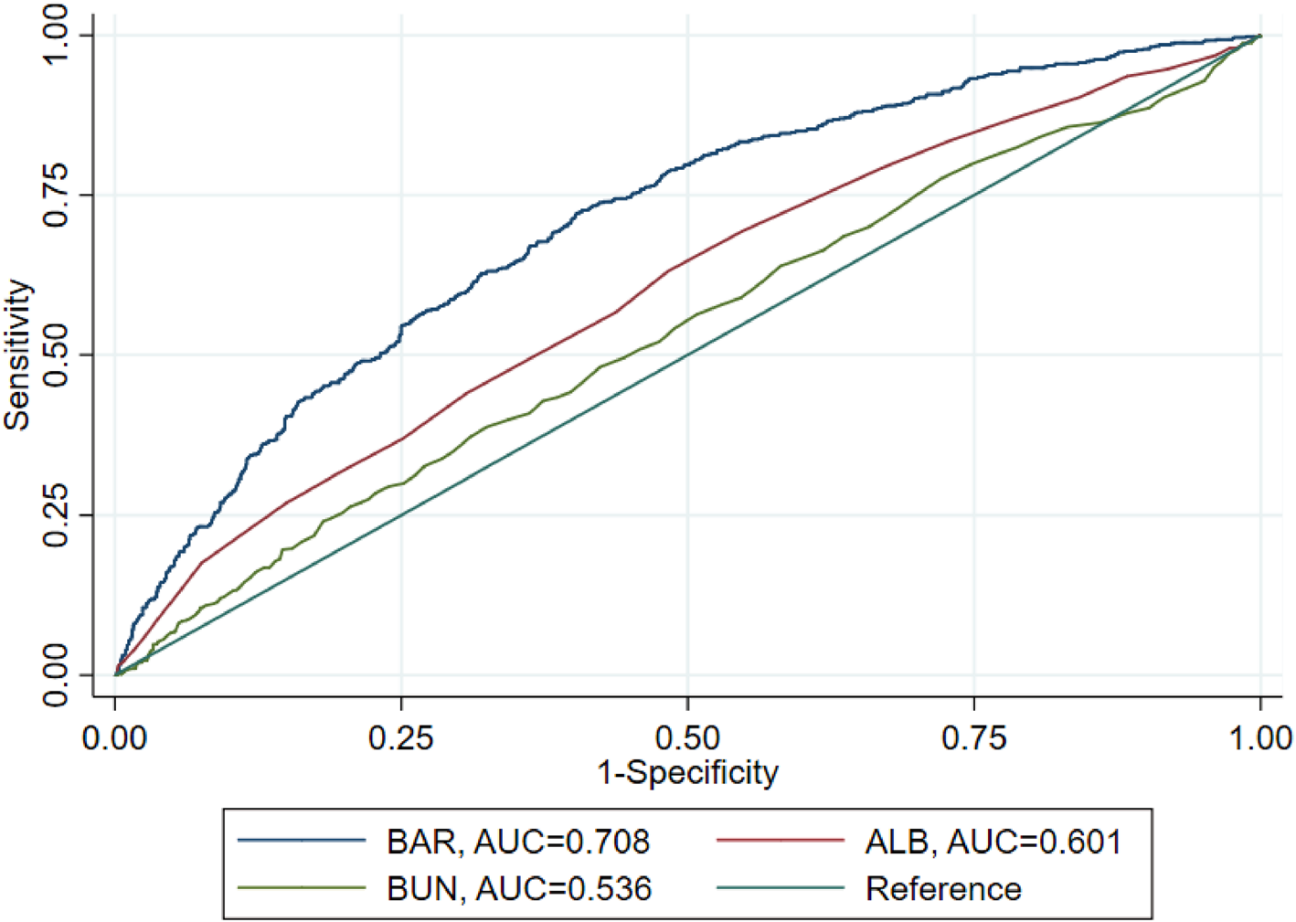
Receiver operating characteristic curves for the prediction of 90-day mortality.

The receiver operating characteristic (ROC) curve generated using the indicator variables (BAR, ALB, and BUN) were shown in Fig. 2. The AUC value of BAR was 0.708, which showed significantly higher AUC value than the ALB and BUN (*p* < 0.05). Similarly, the c-statistic of BUN, ALB, and BAR showed that BAR had the highest c-statistic (Supplementary Table 4).

### Subgroup analyses

Subgroup analyses were used to determine the consistency of association between BAR and risk of 90-day mortality (Table 5). Subgroup analyses showed that patients with age > 75(HR 1.024, 95% CI 1.013–1.034), man (HR 1.022, 95% CI 1.012–1.032), hypertension (HR 1.023, 95% CI 1.010–1.037), CHF (HR 1.019, 95% CI 1.008–1.030), CAD (HR 1.022, 95% CI 1.007–1.038), SOFA score ≥ 5 (HR 1.014, 95% CI 1.005–1.023), weight ≥ 77 (HR 1.027, 95% CI 1.016–1.038), CKD 3 stage (HR 1.023, 95% CI 1.011–1.035), phosphate < 4 (HR 1.019, 95% CI 1.005–1.033), WBC ≥ 11.3 (HR 1.019, 95% CI 1.010–1.029), lactate ≥ 2.4 (HR 1.024, 95% CI 1.013–1.035), ALP ≥ 103 (HR 1.022, 95% CI 1.012–1.032) and PaO_2_ < 155 (HR 1.019, 95% CI 1.009–1.029) had a significantly higher risk of 90-day mortality with high BAR level. The results also showed that high BAR was significantly associated with increased 90-day mortality in these patients with CKD stage 5 (Supplementary Table 5).

**Table 5:** Subgroup analysis of the associations between BAR and 90-day mortality.

Subgroup	HR (95%CI)	*P* value
Age, years		
< 75	1.022 (1.011–1.034)	< 0.001
≥ 75	1.024 (1.013–1.034)	< 0.001
Gender		
F	1.014 (1.003–1.026)	0.013
M	1.022 (1.012–1.032)	< 0.001
Hypertension		
No	1.018 (1.008–1.027)	< 0.001
Yes	1.023 (1.010–1.037)	0.001
CHF		
No	1.019 (1. 008–1.029)	< 0.001
Yes	1.019 (1.008–1.030)	0.001
CAD		
No	1.017 (1.009–1.026)	< 0.001
Yes	1.022 (1.007–1.038)	0.005
SOFA score		
< 5	1.013 (0.996–1.031)	0.130
≥ 5	1.014 (1.005–1.023)	0.001
Weight		
< 77	1.017 (1.006–1.028)	0.003
≥ 77	1.027 (1.016–1.038)	< 0.001
CKD stage		
3	1.023 (1.011–1.035)	< 0.001
4	1.022 (1.009–1.035)	0.001
5	1.019 (1.006–1.032)	0.040
WBC		
< 11.3	1.014 (1.002–1.027)	0.023
≥ 11.3	1.019 (1.010–1.029)	< 0.001
Phosphate		
< 4	1.019 (1.005–1.033)	0.008
≥ 4	1.017 (1.007–1.027)	< 0.001
Lactate		
< 2.4	1.013 (1.003–1.024)	0.014
≥ 2.4	1.024 (1.013–1.035)	< 0.001
PaO_2_		
< 155	1.019 (1.009–1.029)	< 0.001
≥ 155	1.014 (1.001–1.026)	0.028
ALP		
< 103	1.013 (1.001–1.025)	0.041
≥ 103	1.022 (1.012–1.032)	< 0.001

### Kaplan–Meier analysis

**Figure 3:**
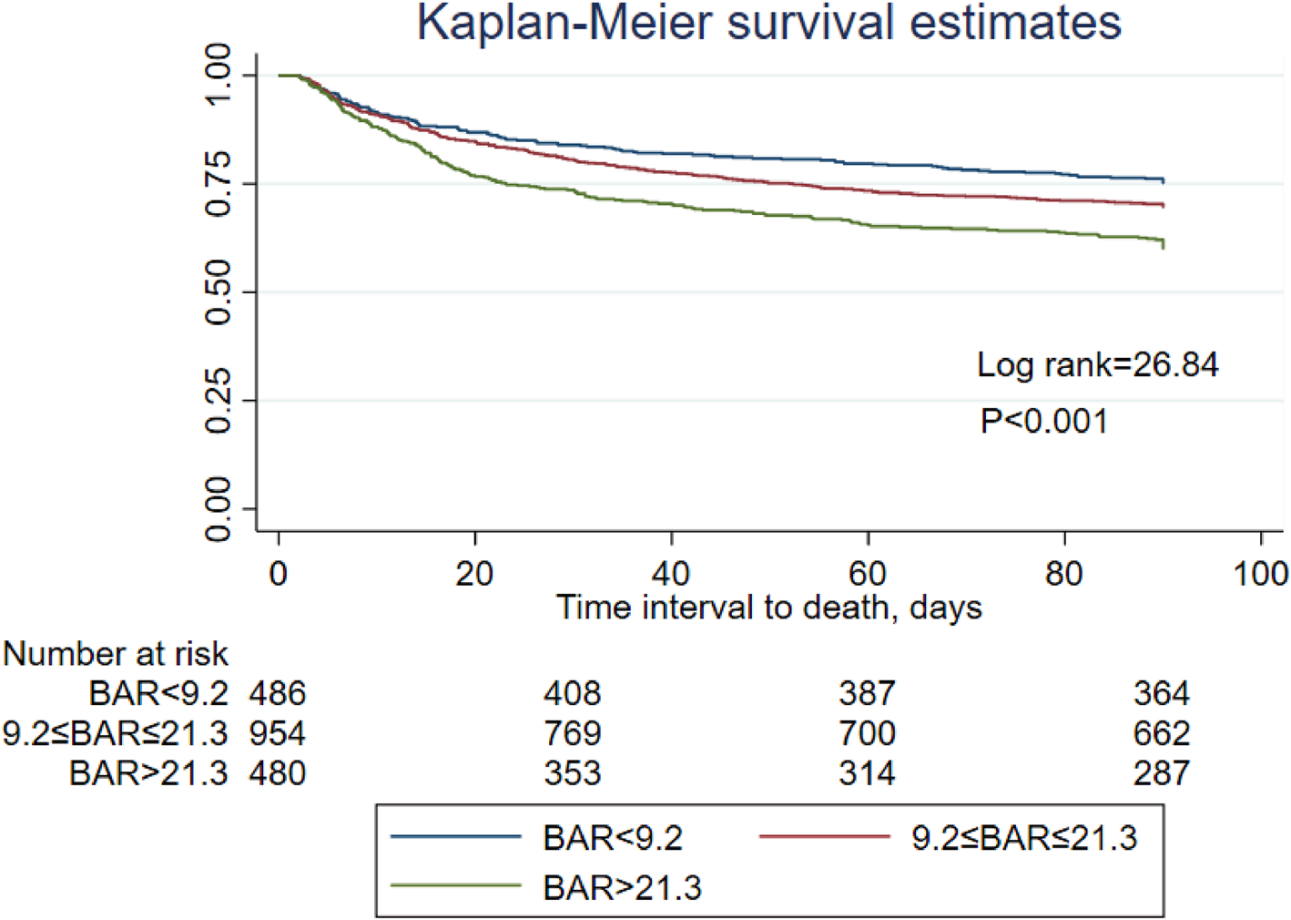
Kaplan–Meier curve was used to evaluate the difference between BAR levels and 90-day mortality in T2DM patients with CKD in the ICU. In the Kaplan–Meier analysis, the log-rank test *P* value < 0.001.

The Kaplan–Meier survival curve analysis showed that BAR > 21.3 had worst prognosis. Patients in higher BAR group had significantly higher risk of 90-day mortality than low and mid-BAR groups (BAR > 21.3 vs 9.2 ≤ BAR ≤ 21.3 vs BAR < 9.2; 40.2% vs 30.6% vs 25.1%, respectively; log-rank test p value < 0.001) (Fig. 3).

## Discussion

With the increasing prevalence of T2DM and CKD in worldwide, timely identification of prognostic risk factor is particularly important in clinical work. In our study, we found that higher BAR on admission to ICU was significantly associated with an increased risk of 90-day mortality in T2DM patients with CKD. And BAR could serve as an independent predictive factor of 90-day mortality. Further, the K-M curve also presented that the high BAR group had a worse prognosis. Our study was the first largest study to explored the relationship between BAR and prognosis in T2DM patients with CKD in the ICU.

BUN is a nitrogen-containing compound that it is influenced by renal function, neurohormone, and sympathetic nervous activity. BUN is mainly filtered through the glomeruli and excreted through urine. When glomerular filtration function decreases, BUN concentration will increase. BUN can not only be used to estimate glomerular filtration function, but also to assess the body's nutritional status, low blood volume, protein metabolism and others^8^. Many studies have found that BUN was a powerful predictor of prognosis in patients with heart failure, and its efficacy was even better than GFR and serum creatinine^16,17^. A large study in China found that BUN levels were positively associated with the risk of developing T2DM in Chinese adults^18^. Studies in T2DM patients showed that the increased BUN level will significantly increase the risk of diabetes retinopathy and diabetes nephropathy^19,20^. Elevated BUN indicated poor prognosis for patients in the ICU^13,21,22^. In the ICU, T2DM patients with CKD had circulatory dysfunction and neuroendocrine system dysfunction, which further aggravated kidney injury. At this time, high levels of BNU may predict a worse prognosis for patients.

ALB is not only a nutritional marker, but also plays an important role in anti-inflammatory, antioxidant and others aspects^23^. As an important antioxidant in plasma, ALB inhibits apoptosis of renal tubular cells by clearing oxygen free radicals^24^. It was found that ALB not only improves renal perfusion and glomerular filtration by prolonging renal vasodilation, but also selectively inhibits the expression of tumor necrosis factor-α-induced vascular cell adhesion molecule 1 and the activation of nuclear factor kB and monocyte adhesion in human endothelial cells to prevent kidney injury^13,25^.In T2DM patients, ALB level was negatively correlated with the incidence of diabetes retinopathy^26^. Besides, a study also showed that hypoproteinemia significantly accelerated the risk of renal failure in patients with diabetes nephropathy^27^. Low ALB level was caused by insufficient nutrition intake and a state of inflammatory stress in ICU patients. Numerous studies had suggested that hypoalbuminemia was a risk factor for poor prognosis in ICU patients^28–30^.

High BAR levels are caused by high BUN or low ALB. BAR has been proven to be a more reliable predictor than BUN or ALB. In our study, ROC curve also showed the AUC value of BAR was significantly higher than the ALB and BUN. Studies suggest that high BAR can significantly increase the mortality of patients with sepsis, acute myocardial infarction, acute pulmonary embolism, heart failure and others^11,21,31,32^. A study found that high BAR significantly increased in-hospital mortality and the incidence of AKI for patients with cerebral hemorrhage in the ICU^13^. Our study also found similar results. We found that when BAR > 21.3, patients' stay in the ICU, hospital mortality, and 30-day and 90-day mortality were significantly increased. After adjusting for confounders such as serum creatinine, CKD and others, elevated BAR was still positively associated with poor prognosis in patients undergoing cardiac surgery^33^. Similarly, in our study, after controlled for confounding variables by multivariate Cox regression analysis, we found that high BAR was an independent risk factor for 90-day mortality. Besides, in subgroup analysis, we demonstrated that BAR was an effective predictor of 90-day mortality in T2DM patients with CKD under various specific conditions. Therefore, in clinical practice, we may be able to reduce the BUN value by improving glomerular filtration rate (such as increasing renal perfusion by maintaining normal volume load), and also by increasing albumin (infusion of human serum albumin, etc.) to reduce BAR, thereby reducing the risk of 90 day mortality in patients. In routine clinical practice, BAR could be calculated easily and quickly, and is more stable and conducive to clinical application compared with a single indicator.

In our study, several limitations should be highlighted to interpret the results as follows: First, this study as a single-centric retrospective study, we couldn’t avoid to selection bias. Second, the data used in the manuscript were recorded from 2001 to 2012, which were old. Third, due to the limited contents of this database, some unrecorded clinical information was missing, may affect the outcome. Fourth, this study only included BUN and albumin records of patients at their first admission, so that the prognostic impact of dynamic changes in BAR was still unclear. Finally, the underlying mechanism of BAR affected the prognosis could not be determined. Therefore, it is necessary to establish a large multicenter prospective study to confirm the above results and further to investigate the mechanism.

## Conclusion

A higher BAR was significantly associated with an increased risk of 90-day mortality. BAR could be served as a prognostic predictor for 90-day mortality in T2DM patients with CKD in ICU, due to its inexpensive and readily available nature.

### Supplementary Information

Supplementary Table 1.

Supplementary Table 2.

Supplementary Table 3.

Supplementary Table 4.

Supplementary Table 5.

## Data Availability

Original data used in this study is from the MIMIC-III database: MIMIC III (https://physionet.org/content/mimiciii/1.4/, version 1.4). The author (S.L.) obtained access to this database (certification number: 42883491) and was responsible for extracting the data. If needed, related data can be provided by contacting G.H. and S.L.
