# Supplementary material for: Blood urea nitrogen to serum albumin ratio as a new prognostic indicator in type 2 diabetes mellitus patients with chronic kidney disease
Source: Sci Rep. 2024 Apr 5;14:8002. doi: 10.1038/s41598-024-58678-4 (PMC10997773; doi:10.1038/s41598-024-58678-4)
Supplement: Supplementary file 1 — Supplementary Table 1. [file 41598_2024_58678_MOESM1_ESM.docx]

Supplementary Table 1. Top five diagnosed diseases in T2DM patients with CKD at admission to ICU.

| ICD_code | Clinical diagnosis | Number |
| --- | --- | --- |
| 25000 | DMII wo cmp nt st uncntr | 1204 |
| 42820 | Systolic hrt failure NOS | 899 |
| 5849 | Kidney failure NOS | 834 |
| 41401 | AMI inferior wall, init | 537 |
| 2724 | Hyperlipidemia NEC/NOS | 430 |
