# Supplementary material for: Blood urea nitrogen to serum albumin ratio as a new prognostic indicator in type 2 diabetes mellitus patients with chronic kidney disease
Source: Sci Rep. 2024 Apr 5;14:8002. doi: 10.1038/s41598-024-58678-4 (PMC10997773; doi:10.1038/s41598-024-58678-4)
Supplement: Supplementary file 2 — Supplementary Table 2. [file 41598_2024_58678_MOESM2_ESM.docx]

Supplementary Table 2. Top five diseases that were first diagnosed in T2DM patients with CKD at admission to ICU.

| ICD_code | Clinical diagnosis | Number |
| --- | --- | --- |
| 0389 | Septicemia NOS | 156 |
| 41071 | Subendo infarct, initial | 112 |
| 5849 | Kidney failure NOS | 58 |
| 41401 | AMI inferior wall, init | 50 |
| 03842 | E coli septicemia | 40 |
