# Supplementary material for: Blood urea nitrogen to serum albumin ratio as a new prognostic indicator in type 2 diabetes mellitus patients with chronic kidney disease
Source: Sci Rep. 2024 Apr 5;14:8002. doi: 10.1038/s41598-024-58678-4 (PMC10997773; doi:10.1038/s41598-024-58678-4)
Supplement: Supplementary file 3 — Supplementary Table 3. [file 41598_2024_58678_MOESM3_ESM.docx]

Supplementary Table 3. Some diseases that might influence the prognosis and clinical data itself.

| Clinical diagnosis | Number |
| --- | --- |
| trauma | 37 |
| burning | 28 |
| intoxication | 34 |
| sepsis | 322 |
| cardiovascular disease | 635 |
| GI tract bleeding | 136 |
