# Supplementary material for: Blood urea nitrogen to serum albumin ratio as a new prognostic indicator in type 2 diabetes mellitus patients with chronic kidney disease
Source: Sci Rep. 2024 Apr 5;14:8002. doi: 10.1038/s41598-024-58678-4 (PMC10997773; doi:10.1038/s41598-024-58678-4)
Supplement: Supplementary file 4 — Supplementary Table 4. [file 41598_2024_58678_MOESM4_ESM.docx]

Supplementary Table 4. The c-statistic of BUN, ALB, and BAR for 90-day mortality.

| Model | C-statistic (95% CI) | P-value |
| --- | --- | --- |
| BAR | 0.708(0.683, 0.732) | Ref. |
| ALB | 0.601(0.574, 0.629) | <0.001 |
| BUN | 0.536( 0.508, 0.564) | <0.001 |
