# Supplementary material for: Blood urea nitrogen to serum albumin ratio as a new prognostic indicator in type 2 diabetes mellitus patients with chronic kidney disease
Source: Sci Rep. 2024 Apr 5;14:8002. doi: 10.1038/s41598-024-58678-4 (PMC10997773; doi:10.1038/s41598-024-58678-4)
Supplement: Supplementary file 5 — Supplementary Table 5. [file 41598_2024_58678_MOESM5_ESM.docx]

Supplementary Table 5. BAR level and clinical outcome in CKD 5 stage.

| Clinical Outcomes | BAR | | | |
| --- | --- | --- | --- | --- |
|  | <12.6(n=56) | 9.2-21.3(n=115) | >30.9(n=56) | P value |
| Length of stay (days) | 2.1(1.1, 4.2) | 2.9(1.4, 5.7) | 2.9(1.3, 5.9) | 0.417 |
| Hospital mortality (n, %) | 10(17.8) | 21(18.3) | 13(23.2) | 0.020 |
| 30-day mortality (n, %) | 12(21.4) | 29(25.2) | 18(32.1) | 0.002 |
| 90-day mortality (n, %) | 15 (26.8) | 37(32.2) | 24(42.9) | <0.001 |
